# 2279: The effects of autoimmune inflammation on proliferation, differentiation, and androgen receptor signaling in adult prostate stem cells

**DOI:** 10.1017/cts.2017.216

**Published:** 2018-05-10

**Authors:** Paula Cooper, Hsing-Hui Wang, Meaghan Broman, Hristos Kaimakliotis, Bennett Elzey, Scott Crist, Liang Cheng, Timothy Ratliff

## Abstract

OBJECTIVES/SPECIFIC AIMS: The primary goal of this project is to verify murine findings in the human setting. METHODS/STUDY POPULATION: The methods include primary cell isolation and culture, FACS, adoptive transfer, 3D-cell culture, histology, immunofluorescence, xenograft, and tissue recombination. The study population includes patients undergoing radical prostatectomy due to hyperplasia or adjacent bladder or prostate cancer. RESULTS/ANTICIPATED RESULTS: Having verified similar sensitivities to androgen receptor (AR) inhibitors between naive murine and human basal prostate stem cells, we anticipate that autoimmune inflammation in humans affects the response of basal prostate stem cells in a manner similar to the murine setting as well. This includes increased proliferation, differentiation, and response to AR inhibitors. DISCUSSION/SIGNIFICANCE OF IMPACT: The identification of survival mechanisms used by basal prostate stem cells in an androgen deprived environment may give insight to the process by which prostate cancer becomes androgen independent. The effect of inflammation on proliferation, survival, and AR signaling in these cells may also provide information relevant to cancer initiation and progression.

